# Dark Side or Bright Side: The Impact of Alcohol Drinking on the Trust of Chinese Rural Residents

**DOI:** 10.3390/ijerph19105924

**Published:** 2022-05-13

**Authors:** Jie Dong, Qiran Zhao, Yanjun Ren

**Affiliations:** 1 School of Economics and Business Administration, Chongqing University, Chongqing 400044, China; dongjie@cqu.edu.cn; 2 College of Economics & Management, China Agricultural University, Beijing 100083, China; zhaoqiran@139.com; 3 College of Economics and Management, Northwest A&F University, Yangling, Xianyang 712100, China

**Keywords:** dark side, alcohol drinking, trust

## Abstract

Existing studies have explored the causal effect of social capital on harmful drinking, while the effect of drinking habits on trust is scant. In China, drinking rituals and drinking culture are considered important ways of promoting social interaction and trust, especially in rural areas where traditional culture is stronger. Based on a field survey in rural China in 2019, this paper explores the relationship between drinking habits and trust. First, we found a negative relationship between drinking habits and trust, indicating that those people who drink alcohol are more likely to have a lower trust. Second, we found significant heterogeneity in the effect of alcohol consumption on social trust across various groups. Specifically, the negative effects of alcohol consumption on trust were stronger for the females than for males; drinking alcohol did not reduce the level of trust among the Chinese Communist Party (CCP) in rural China; compared with the Han nationality, we found that the effect of drinking on trust was not significant for the ethnic minority. Third, we observed that the negative effects of alcohol consumption on trust had thresholds across age and income. Among people under 51, the risk of trust from drinking was greater than for those over 51; the negative effect of drinking on residents’ trust was more obvious in low-income families, but not significant in the group with an annual household income of more than CNY 40,000. Our empirical study provides a deeper understanding of drinking culture in rural China from a dialectical perspective.

## 1. Introduction

In China, the wine culture formed from ancient times has long been an indispensable part of Chinese social interaction [1]. Like other East Asian countries, drinking rituals and drinking culture in China are considered to be important ways of promoting social interaction and trust [2]. Chinese people share stories over wine tables, and the relationship gets better when they get tipsy. In modern society, as an important platform for business negotiation, drinking at dinner is often used to maintain good relations between bosses and employees or to promote business cooperation between business partners [3,4,5]. For example, Huang et al. [6] found that male CEOs in areas with a strong drinking culture had more social connections, both inside and outside the company. Hao et al. [7] also believe that social drinking is an essential skill for managers, which can relieve tension and embarrassment and promote social interaction. However, there is not much commercial activity in rural areas, and social drinking is different from that in urban areas. On the one hand, the social capital (social trust and social network) of Chinese rural residents is relatively simple. Drinking is not as utilitarian as commercial drinking tables; on the other hand, the traditional culture in rural areas is more preserved, and the culture of drinking tables during festivals is one of the important ways of maintaining rural social networks. Therefore, the main motivation of this study was to explore the impact of drinking on the social capital or social network of rural residents.

Existing studies have explored the link between social capital and harmful drinking (HD) or alcohol consumption [8,9,10,11]. Moreover, most of these studies focus on areas with drinking habits and cultures. For example, researchers have looked at the impact of alcohol consumption on social relations in northern Europe, where drinking is well known within Europe [12]. Ahnquist et al. [13] found that lack of trust in institutions increased the likelihood of harmful alcohol consumption in Sweden. However, another study from Sweden demonstrated that social capital at the contextual level showed very weak effects on alcohol consumption for teenagers [14]. In Denmark, friends often get drunk as a sign of mutual respect [15]. Similarly, China also has a longstanding drinking culture; for example, there are many ancient Chinese poems related to drinking. In the context of wine culture, drinking is also very popular and common in China. Several studies have examined the impact of social capital on HD in China [16,17,18]. For instance, a study found that a high level of social capital may promote HD among the residents of Chinese neighborhoods [16]. However, taking Chinese migrant workers as the research object, Gao et al. [17] concluded that higher social capital reduces the possibility of problematic drinking among migrant workers. Additionally, in Taiwan, China, Chuang et al. [19] found that social engagement promoted drinking in both men and women. Others focused on adolescents and found that trust is significantly associated with drinking [20,21,22,23,24]. In addition, a large number of studies have found the influence of peer effects of adolescent social interaction on drinking habits [25,26,27,28].

Previous studies analyzed the effect of social capital on alcohol intake—social capital or social trust is the cause. For instance, Gao et al. [17] studied the influence of social capital on problematic drinking among migrant workers in China and found that higher individual-level social capital may protect against HD. However, studies on the effect of drinking habits on social trust are scant. In particular, we do not know how and to what extent alcohol consumption could affect trust among Chinese. Unlike the extensive literature focusing on the health effects of alcohol consumption [29,30,31], the impact of alcohol consumption on social capital has not received much attention. Meanwhile, in recent years, the Chinese government has implemented the “rural revitalization” strategy, which aims to improve the sense of contentment and happiness of rural Chinese. Good social capital and trust relationships are the premises behind enhancing people’s well-being [32]. In rural China, the social relationship among residents is not complicated, and information transmission is not as fast as that between cities (e.g., the Internet and smartphone usage rates are relatively low). As a result, their level of trust also differs from that of urban residents. Moreover, trust can be divided into vertical trust and horizontal trust. Vertical trust—namely, institutional trust—refers to residents’ trust in the institutional environment, involving government credibility in administration, judicature, taxation, and so on; horizontal trust is the general value of non-institutional trust—that is, the trust between friends, relatives, and neighbors in the general sense [33,34,35,36]. In order to make the research more comprehensive, we considered both vertical trust and horizontal trust in the construction of trust indicators.

Based on the above background, our research questions also came out: does drinking affect the trust of rural residents? If so, is it negative (dark side) or positive (bright side)? In addition, is there heterogeneity in the effect among different subgroups?

Based on a field survey in rural China, this research explored the relationship between drinking habits and social trust. The rest of the article is organized as follows: Section 2 deals with theories and hypotheses; Section 3 presents data, variables, and the model; Section 4 presents the regression results; Section 5 discusses the results; and Section 6 closes with our conclusion.

## 2. Literature Review

A large body of literature has also demonstrated that drinking alcohol can enhance the social capital of residents, including social trust and social networks [37,38,39,40,41,42]. Drinking is more of a social culture than a personal habit or preference, and it is deeply ingrained around the world [6,43]. For the most part, drinking is seen as a social lubricant that can be used as a medium to enrich people’s social networks. Some economists argue that drinking can induce people to reveal (unwillingly) information about themselves, thus pulling people into social distancing [44,45,46]. Some studies find that drinking alcohol can promote trust. For example, in studies set in Denmark, increased drinking among adults has been accompanied by an increase in trust [37,38]. Sayette et al. [39] claim that alcohol consumption promotes emotion-related behaviors at the individual and group levels in two ways: it enhances positive behaviors and decreases negative behaviors. Frank et al. [46] found that drinking alcohol does not necessarily mean increased trust, but moderate drinking does. Other studies have found that drinking strengthens social networks. For example, Bray [47] found that moderate drinking increases wage returns and accumulation of social experience and social capital. Similarly, another study, from Germany, showed that alcohol consumption increased wage returns and strengthened social networks [48]. Moreover, Groh et al. [49] from the opposite perspective, found that abstinence harms social networks among friends. In addition, studies have shown that drinking alcohol increases friendships and produces a sense of connection with others [50,51,52]. For example, MacLean [50] suggested that drinking alcohol enhances intimacy and demonstrates trust, especially at higher levels of intoxication, based on interviews of those aged 18–24 years in Australia.

A second branch of the literature shows that alcohol consumption harms social capital. Some studies have shown that alcohol-dependent people have significant emotional empathy deficits that make it difficult for them to trust others [53,54]. Moreover, drinkers tend to fall into a vicious cycle of self-centeredness: drinking leads to self-centeredness, which in turn leads to alcoholism [55,56]. Furthermore, drinking can weaken rationality, and when people are not completely rational in social interactions, lying and cheating can occur [57,58]. Ahnquist et al. [13] found that low levels of institutional trust were associated with an increased likelihood of dangerous alcohol use among adults in Sweden. Similarly, Lindstrom [59] found that alcoholics in Sweden were generally less trusting. Other studies have shown that drinking tends to have negative social effects. For example, Fielding et al. [60] proved that drinking alcohol makes people less generous. Schweitzer et al. [61] conducted a scenario simulation to explore the impact of drinking on personal decision-making and found that drinkers were more likely to make radical choices and make mistakes.

The third branch of studies has found that drinking does not affect people’s social capital. Bregu et al. [62] suggested that alcohol consumption has little systemic effect on economic behavior. From a business perspective, Brañas-Garza et al. [63] found that drinking alcohol does not affect the outcome of negotiations. Another interesting study reports that drinking increased males’ promises to others, but had no effect on their fulfillment—suggesting that drinking does not, at the least, increase people’s trust levels [64].

## 3. Data and Method

### 3.1. Source of Data

The data for this study came from a primary survey in 2019 by the Center for Human Capital and Geoeconomics (CHCG) at China Agricultural University. The survey was approved by the Ethics Committee of China Agricultural University, and a study on the link between alcohol and depression also use these data [65]. This survey covered 50 villages from 7 provinces of mainland China (Heilongjiang, Henan, Zhejiang, Yunnan, Xinjiang, Shandong, Anhui). Ten households were randomly selected from each village. Before data collection, all respondents voluntarily signed an informed consent form after receiving the questionnaire for scientific research. Moreover, they were also told that the feedback was confidential. After matching all the variables and dropping observations with missing covariates, the final sample consisted of 5207 rural adults.

### 3.2. Variables

#### 3.2.1. Dependent Variable

The explained variable of this research was the trust level of rural residents. Trust can be divided into vertical trust and horizontal trust [33]. Vertical trust represents institutional trust, which refers to residents’ trust in the institutional environment, involving government credibility; horizontal trust is the general value of non-institutional trust—that is, the trust between friends, relatives, and neighbors in the general sense [34]. Therefore, we also considered both vertical trust and horizontal trust in the design of the questionnaire. In China’s rural areas, village cadres are the policy transmitters and managers that villagers contact directly, so the trust of village cadres to a large extent represents the vertical trust of residents. Horizontal trust includes the trust of neighbors, kin, and friends, which are the most common social objects in rural China. In the questionnaire, we designed four indexes about the trust degree both from vertical trust and horizontal trust: the trust degree of village cadres (vertical trust), neighbors, kin, and friends (horizontal trust). As shown in Table 1, each trust indicator for trust levels from lowest to highest is presented on a scale of 1–10.

To better quantify the trust degree of villagers, we used principal component analysis (PCA) to reduce the dimensionality of each trust variable, while minimizing the loss of information. First, we examined whether the data support PCA. As shown in Table 2, the KMO value was 0.738 (higher than the threshold of 0.7), indicating that the data support the PCA method. Moreover, we see that only the eigenvalue of Comp1 is 2.330, greater than 1, indicating that the Comp1 can be used as a linear combination of variables.

**Figure 1:**
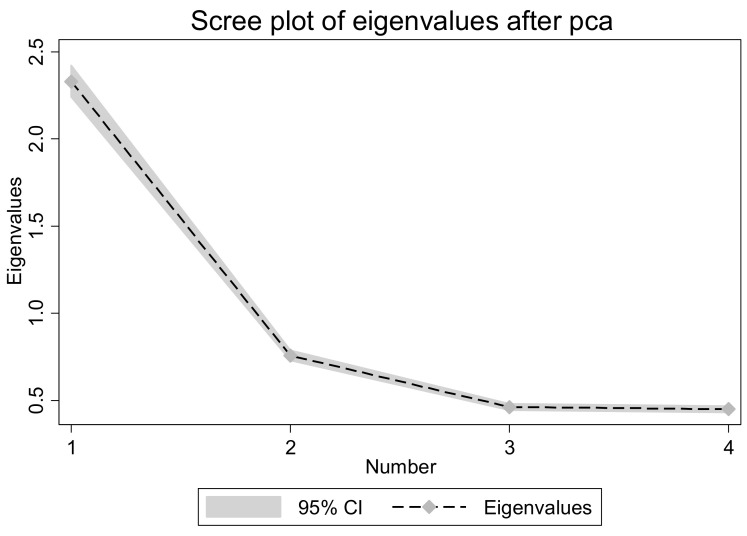
Scree plot of PCA.

To further prove that Comp1 is the only principal component, we also drew a scree plot. As shown in Figure 1, the abscissa represents the number of principal components and the ordinate represents the eigenvalues. When the x-coordinate exceeds 2, the eigenvalues begin to flatten out, so it is appropriate to choose an eigenvalue. In conclusion, it is convincing to choose Comp1 as the principal component of the variable.

**Figure 2:**
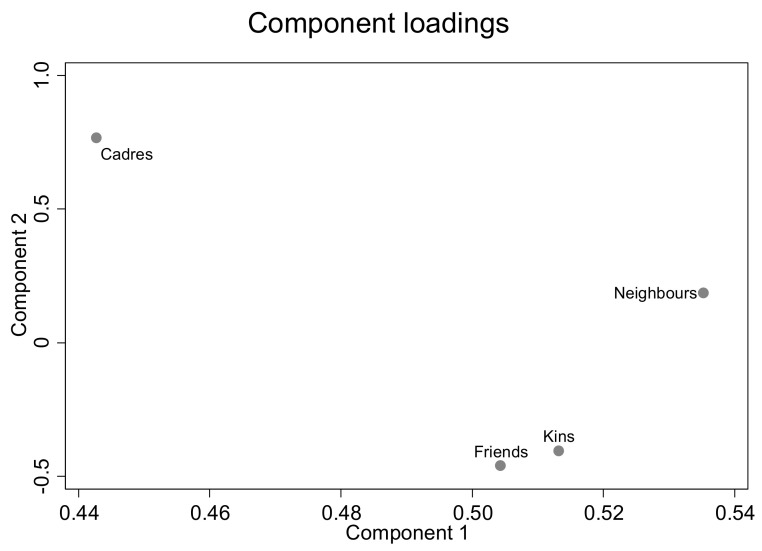
Loading diagram for PCA.

Finally, we have the loading value of Comp1, as shown in the abscissa of Figure 2, which can intuitively present the impact of each variable on the principal component, which is, according to the loading value in ascending order: village cadres (0.443), friends (0.504), kin (0.513), and neighbors (0.535). Thus, we achieved the purpose of dimensionality reduction for the four trust variables and obtained a composite indicator, which is the variable Trust. The statistic of the Trust variable is shown in Table 1.

#### 3.2.2. Independent Variable

The core independent variable of this study was whether residents drink alcohol and was defined as “*B_drink*”. In the field study, the question was “Do you currently drink alcohol?” Participants responded with “Yes = 1; No = 0”. The core independent variable of this study was whether residents drink alcohol. As shown in Table 1, the proportion of rural residents who drink alcohol was close to 70%, indicating that the proportion of rural residents who drink alcohol was relatively high in rural China.

#### 3.2.3. Control Variables

This research added control variables according to the ecological model [66]. In our case, factors affecting residents’ trust come from five dimensions: individual factors, interpersonal factors, organizational factors, community factors, and public policy factors. This paper first controlled individual factors (gender, ethnicity, age, education, health) [20,67,68]. Second, we used the number of friends and whether respondents used the social APP WeChat as interpersonal variables. Third, we controlled for organizational factor variables: whether the family was engaged in agriculture and the number of family members. Fourth, we added the two distance variables as community factors: distance from household to village committee and distance from household to county center. The closer they lived to the village committee, the wider their social network within the community. In addition, the closer the villager was to the county center, the more extensive and the faster the information they received. Last, we used whether residents were Chinese Communist Party (CCP) members and whether they cared about political news as public policy factors. Because in rural China members of the CCP often serve as village officials, they tend to have a broader social network [69,70]. The definition and statistics of control variables are shown in Table 1.

### 3.3. Model and Preliminary Statistical Analysis

#### 3.3.1. Model

The following econometric model was estimated:

(1) $$Trust_{i}=\alpha +\beta B\_drink_{i}+\gamma X_{i}+\varepsilon_{i}$$

where the subscript i indicates the individuals; *Trust* is the comprehensive trust index of residents by PCA; *B_drink* is the core explanatory variable based on the question “Do you currently drink alcohol?” and replying “yes = 1, no = 0”. *X* is a vector of variables that controls the five dimensions of the ecological model mentioned above; ξ is a random disturbance term.

#### 3.3.2. Preliminary Statistical Analysis

As shown in Table 3, we divided the residents into drinking samples and non-drinking samples to observe the statistical differences between the two groups. It can be seen that the explained variable *Trust* in this paper had a difference of 0.203 between the two groups and was significant at the 5% level, which means that residents who do not drink have higher levels of trust.

## 4. Results

### 4.1. Baseline Results

The effect of drinking on trust is shown in Table 4. In Column 1, the coefficient of drinking for individuals is −0.203 at a significant 1% level. Adjusting for individual factors (Column 2), the association between drinking and trust is strengthened, with a coefficient value of −0.409, which is significant at the 1% level. The effect of drinking on trust is also significantly decreased with additional *Friends* and *WeChat* variables (Column 3). It shows that the negative effect of drinking on trust still exists after controlling for the confounding interpersonal factors. After controlling organizational factors, the coefficient of the core explanatory variable is −0.350 and is significant at the 1% level (Column 4). After the community factors are added, the core explanatory variable decreases to −0.320, but it is still significant at the 1% level (Column 5). Finally, after controlling all variables, the coefficient of the core explanatory variable is −0.354, which is significant at the level of 1%, indicating that drinking has a negative relationship with residents’ trust with full control.

### 4.2. Identifying Causal Effects

In the baseline regression model, the main source of endogeneity problems is reverse causality. To deal with this endogeneity, we employed the technique by Lewbel [71] to identify causality. The products of exogenous covariance and heteroscedastic error can be used as effective instrumental variables to identify endogenous parameters when effective instrumental variables cannot be found. In our case, we used the following equations:

(2) $$Trust=\alpha_{1}X+\beta_{1}B\_drink+\varepsilon_{1}$$

(3) $$B\_drink=\alpha_{2}X+\varepsilon_{2}$$

where $\beta_{1}$ is the parameter of the core independent variable, B_drink is the endogenous variable, and X is a vector of control variables. We assumed there was not a valid IV for B_drink and that the error $\varepsilon_{2}$ was heteroscedastic—that is, $\mathrm{Cov}\left(X,\varepsilon_{2}^{2}\right)\neq 0$. According to the deduction, $\left(X-\bar{X}\right)\varepsilon_{2}$ would be the valid IV for B_drink.

Table 5 reports the results of the regression using Lewbel’s method. Column 1 is the first-stage regression, and column 2 is the second-stage regression. First, we found that the null hypothesis of homoscedasticity was strongly rejected (the chi-squared statistic was 113.10, at a 1% significance level), conforming to the prerequisites of the method. Second, the coefficient was −0.701 at a 10% level of significance. This indicates that drinking alcohol does have a decreasing effect on trust.

### 4.3. Heterogeneity Analysis

#### 4.3.1. Heterogeneity of Gender, Political Status, and Ethnicity

**Figure 3:**
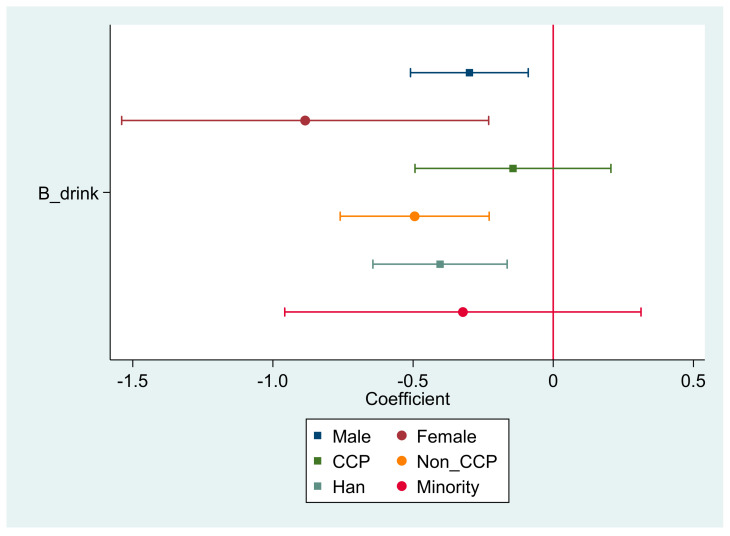
Heterogeneity analysis.

To further explore whether the effects of drinking on trust differ in different groups, Figure 3 reports the heterogeneity of the effects of drinking on trust. As shown in the first two lines of Figure 3, the effect of drinking on trust is significantly different between men and women. The negative effect of drinking on trust is much greater in women than in men, and both are significant at the level of 1%. In Chinese society, especially in the relatively conservative rural areas, there is some social pressure for women to drink [72,73]. As a result, if a woman drinks, she experiences more social stress, is less likely to engage in social activities, and has lower levels of trust.

Then, we focused on the heterogeneity of the effect between CCP members and non-CCP members and found that drinking had no significant effect on trust among CCP members, while this negative effect passed the statistical test among non-CCP members. The results suggest that drinking does not reduce trust among CCP members. Finally, the effect of alcohol consumption on trust was observed to be heterogeneous across ethnic groups. In the Han population, drinking decreases villagers’ trust, but in the minority population, drinking does not decrease people’s trust (did not pass the statistical test).

#### 4.3.2. Threshold Heterogeneity Analysis on Age and Income

Are the effects of alcohol consumption on trust linear across all age and income groups? Is there a threshold at which the effect shifts on either side of the threshold? If so, what is the threshold? Hansen [74] pioneers the threshold regression model to explore the phenomenon that when one economic parameter reaches a certain value, another economic parameter suddenly shifts to other forms of development (structural mutation). In this research, the threshold regression models were used to observe the regression results of different age groups and different income groups. The threshold regression results are reported in Table 6. Columns 1 and 2 report threshold regression results for age, and we found a threshold at age 51. When the age was less than 51 years, the coefficient of the core explanatory variable was −0.489; when the age was more than 51 years, the coefficient of the core explanatory variable was −0.271, both of which were significant at the 1% level. This suggests that although the negative effect of drinking on trust declines across all age groups, the effect of drinking on trust declines significantly after age 51. Columns 3 and 4 report threshold regression results for household income. It can be seen that the influence of drinking on trust exists at a threshold when the family income is CNY 40,000. Alcohol consumption still harms trust when household income is less than CNY 40,000. However, while the family income is more than CNY 40,000, the influence of drinking on trust fails to pass the statistical test, indicating that drinking on trust is not significant.

## 5. Discussion

Why do we conclude that drinking is not good for trust among rural Chinese? First, this research found that one of the main reasons is that Chinese residents’ social demand and social purpose are not strong. As Cooper [75] says, motivation for drinking is important. The purpose of drinking is to foster feelings that lead to some sort of potential deal between superiors and subordinates or business partners [46]. For rural Chinese, however, the motivation for drinking is often not utilitarian. In addition, rural residents do not have much need for social contact; except in traditional festivals or weddings and funerals, Chinese rural residents usually drink alone. As a result, rural Chinese do not gain trust through drinking. In contrast, consistent with our findings, alcohol consumption actually impairs trust. Especially in the context of China’s large inflow of rural labor force into cities, the proportion of elderly people left behind in rural areas is large, and these people often drink alone. Some studies have shown the negative effects of drinking alone on social capital [76,77,78]. For example, Yu et al. [79] claim that among elderly people living alone in rural China, drinking alone can create feelings of distrust. Second, drinking lowers people’s rational boundaries and makes them more likely to say exaggerated things, causing distrust. Contrary to some opinions, people are more likely to lie when under the influence of alcohol [80,81,82]. A recent study from China also found a significant increase in lying about the effects of alcohol in business negotiations [58]. In China’s drinking culture, people tend to exaggerate and make empty promises. Hence, drinking reduces trust among residents. The third reason is that in Chinese culture, people who gather to drink often get drunk, and not getting drunk at the wine table is considered to be insincere. There is a Chinese saying, “If you don’t get drunk tonight, you won’t return.” Therefore, in rural China, once residents drink together, it is highly likely to cause drunkenness. However, alcoholism and drunkenness are considered not only harmful to the drinkers’ health but also have many negative effects on others. For example, drinking is a breeding ground for domestic violence. A study from Mexico found that in rural areas, men who drank alcohol often used domestic violence against their wives and that it was common there [83]. In rural communities, many residents have expressed concern about the disruption of alcohol consumption to quiet daily life and the well-being of individuals, families, and society. Alcohol abuse causes people in the same community to distrust alcoholics, and the distrust is mutual, making drinkers less trusting of others. The last reason is that drinking alcohol can lead to drunk driving, which is illegal in China. On the one hand, drunk driving increases the incidence of traffic accidents and damages the lives of residents. In China, on the other hand, drivers who have ingested even small amounts of alcohol will face revocation of their license and fines if caught by traffic police. As a result, their record of breaking the law makes them less trusted. Correspondingly, their trust is also reduced.

In addition, we observed heterogeneity across subgroups in the perception of trust from drinking to residents. One finding is that the negative effects of alcohol consumption on trust were stronger in the female subgroup than in the male subgroup. This is also easier to explain because women are traditionally frowned upon in culture for drinking alcohol. In some areas, women who drink alcohol are labeled as having weak volition and otherwise labeled negatively [84]. Especially in rural China, where thinking is relatively traditional, women drinking alcohol is also considered to be immoral to a certain extent [72]. Lack of respect and understanding for women’s drinking has also led to a significant drop in trust among women. Second, we found that drinking alcohol does not reduce the level of trust among CCP in rural China. The possible reason is that in rural China, CCP members are generally local village cadres or rural elites, and their social network itself is relatively stable, so drinking does not have a strong negative impact on trust (although the regression sign was also negative, it was not significant). Moreover, CCP members themselves believe in materialism and have strong political beliefs. Third, compared with the Han nationality, we found that the influence of minority drinking on trust was not significant. Ethnic minorities like to drink more than Han people, such as Tibetans and Mongolians. Drinking is an essential medium in their culture for making friends [72]. Therefore, for ethnic minorities, the negative impact of trust on them is not significant.

The negative effects of alcohol on trust jump around the age of 50—the effect decreases significantly after that age. On the one hand, as one study of China shows, older people have a greater sense of community and trust in government than younger people [85]. On the other hand, after the age of 50, people have more social experience and insights into societal risks than when they were young, and they have a deep understanding of friendship and kinship [86]. Especially in rural China, clans and traditional culture mean that the elderly people have more say [87]. As a result, drinking is less damaging to older adults’ trust. In addition, we found that the negative effect of alcohol consumption on trust was significant in the lower-income group but not in the higher-income group. Research shows that socioeconomic status (as measured by income) significantly improves residents’ social trust [88]. A study from Russia also demonstrated a positive causal relationship between economics and trust through a natural experiment [89]. Therefore, the trust level is higher in high-income families, and the negative effect of drinking on their trust is not obvious. In contrast, alcohol consumption significantly reduces the trust of low-income families.

## 6. Conclusions

This paper discusses the relationship between drinking and trust based on primary survey data in rural China. Although previous literature holds different attitudes towards the influence of alcohol on individual social capital, our study found that drinking shows its dark side to residents’ trust.

First, we found a negative relationship between drinking habits and trust, where people who drink alcohol are more likely to lower their trust. Second, we focused on the heterogeneity of the effect of alcohol consumption on trust in different groups: the negative effects of alcohol consumption on trust were stronger in the female subgroup than in the male subgroup; drinking alcohol did not reduce the level of trust among CCP in rural China; compared with the Han nationality, we found that the influence of minority drinking on trust was not significant. Third, we observed that the negative effects of alcohol consumption on trust have thresholds across age and income. Among people under 51, the risk of trust from drinking was greater than for those over 51; the negative effect of drinking on residents’ trust was more obvious in low-income families, but it was not significant in the group with an annual household income of more than CNY 40,000.

Our empirical study provides a deeper understanding of drinking culture in rural China from a social trust perspective. Policymakers need to highlight not only the health risks of drinking but also the damage it does to trust. For drinkers, it is important to recognize that drinking does not benefit social capital but that it creates a crisis of trust.

## Figures and Tables

**Table 1:** Definition and statistics of variables.

Variable	Variable Definitions	Mean	S.D.	Min	Max
***Dependent variable***					
Trust in cadres	The degree of trust to village cadres: 0–10; low–high	8.712	1.973	0	10
Trust in neighbors	The degree of trust to neighbors: 0–10; low–high	8.246	1.805	0	10
Trust in kin	The degree of trust to kin: 0–10; low–high	9.079	1.351	0	10
Trust in friends	The degree of trust to friends: 0–10; low–high	8.643	1.832	0	10
Trust	Comprehensive indicators of trust obtained by PCA	17.286	2.640	1.934	19.950
***Independent variables***					
*B_drink*	Do you currently drink alcohol? 1 = yes; 0 = no	0.694	0.461	0	1
***Control variables***					
*Individual factors*					
Gender	1 = male; 0 = female	0.845	0.362	0	1
Ethnic	1 = Han; 0 = minorities	0.822	0.382	0	1
Age	Years	50.618	10.822	19	80
Edc	Education years	7.932	3.340	0	15
Health	Excellent–poor: 1–5	1.956	1.015	1	5
*Interpersonal factors*					
Friends	Number of friends	18.182	10.736	0	100
WeChat	Do you use the social networking APP WeChat? 1 = yes; 0 = no	0.893	0.309	0	1
*Organizational factors*					
Agriculture	Is your family engaged in agriculture? 1 = yes; 0 = no	0.899	0.302	0	1
F_mem	Number of family members	3.934	1.668	1	15
*Community factors*					
Distance_V	Distance from household to village committee (km)	1.590	11.449	0	100
Distance_T	Distance from household to county center (km)	22.710	19.203	0	200
*Public policy factors*					
CCP	Are you a member of the Chinese Communist Party (CCP)? 1 = yes; 0 = no	0.281	0.450	0	1
P_news	Do you follow political news? 1 = yes; 0 = no	7.826	2.811	0	10

**Table 2:** Statistics of principal components.

Component	Eigenvalue	Difference	Proportion	Cumulative
Comp1	2.330	1.573	0.583	0.583
Comp2	0.758	0.295	0.189	0.772
Comp3	0.463	0.013	0.116	0.888
Comp4	0.450	——	0.112	1.000
KMO	0.738			

**Table 3:** Mean differences in *Trust* between drinking and non-drinking.

	Mean of Non-Drinking (ND)	Mean of Drinking (D)	Mean of D-ND
Trust	17.427	17.224	−0.203 **
	(2.667)	(2.625)	[2.565]

Note: Standard deviation in parentheses; T values in square brackets; *** *p* < 0.01, ** *p* < 0.05, * *p* < 0.1.

**Table 4:** The results of baseline regression.

	(1)	(2)	(3)	(4)	(5)	(6)
Variables	Trust	Trust	Trust	Trust	Trust	Trust
*B_drink*	−0.203 *	−0.409 ***	−0.337 **	−0.350 **	−0.320 **	−0.354 ***
	(0.104)	(0.121)	(0.138)	(0.137)	(0.129)	(0.125)
Gender		0.104	0.170	0.181	0.103	−0.081
		(0.188)	(0.205)	(0.204)	(0.192)	(0.194)
Ethnic		0.332 *	0.418 **	0.383 *	0.348 *	0.446 **
		(0.168)	(0.200)	(0.202)	(0.197)	(0.202)
Age		0.007	0.012 **	0.010 *	0.010 *	−0.002
		(0.005)	(0.005)	(0.006)	(0.005)	(0.005)
Edc		0.077 ***	0.073 ***	0.071 ***	0.063 **	0.006
		(0.020)	(0.026)	(0.026)	(0.026)	(0.029)
Health		−0.310 ***	−0.336 ***	−0.349 ***	−0.331 ***	−0.293 ***
		(0.052)	(0.062)	(0.063)	(0.060)	(0.061)
Friends			0.002 ***	0.002 ***	0.002 ***	0.001 ***
			(0.001)	(0.000)	(0.000)	(0.000)
WeChat			0.037	0.031	0.031	−0.007
			(0.217)	(0.218)	(0.207)	(0.206)
Agriculture				−0.107	−0.119	−0.127
				(0.192)	(0.194)	(0.191)
F_mem				−0.076	−0.071	−0.077
				(0.049)	(0.049)	(0.050)
Distance_V					0.003 *	0.003 *
					(0.002)	(0.002)
Distance_T					−0.002	−0.001
					(0.004)	(0.004)
CCP						0.411 ***
						(0.121)
P_news						0.142 ***
						(0.025)
Constant	17.427 ***	16.840 ***	16.466 ***	17.037 ***	17.205 ***	17.084 ***
	(0.103)	(0.319)	(0.483)	(0.601)	(0.592)	(0.616)
Observations	5207	5207	5207	5207	5207	5207
R-squared	0.001	0.030	0.034	0.036	0.032	0.058

Note: Standard errors in parentheses (clustered at the county level). *** *p* < 0.01, ** *p* < 0.05, * *p* < 0.1.

**Table 5:** Identifying causal effects based on Lewbel’s method.

	(1)	(2)
Variables	*B_drink*	Trust
*B_drink*		−0.701 *
		(0.398)
Gender		−0.084
		(0.194)
Ethnic		0.441 **
		(0.201)
Age		−0.001
		(0.006)
Edc		0.003
		(0.031)
Health		−0.291 ***
		(0.061)
Friends		0.001 ***
		(0.000)
WeChat		0.014
		(0.208)
Agriculture		−0.125
		(0.190)
F_mem		−0.078
		(0.050)
Distance_V		0.003 *
		(0.002)
Distance_T		−0.002
		(0.004)
CCP		0.414 ***
		(0.121)
P_news		0.141 ***
		(0.024)
Error*c_Gender	−0.471	
	(0.314)	
Error*c_Ethnic	0.470	
	(0.723)	
Error*c_Age	−0.051 ***	
	(0.016)	
Error*c_Edc	0.126 ***	
	(0.047)	
Error*c_Health	−0.018	
	(0.113)	
Error*c_Friends	0.001	
	(0.002)	
Error*c_WeChat	−1.118 ***	
	(0.317)	
Error*c_Agriculture	0.169	
	(0.517)	
Error*c_F_mem	0.049	
	(0.100)	
Error*c_Distance_V	−0.006	
	(0.007)	
Error*c_Distance_T	0.004	
	(0.013)	
Error*c_CCP	−0.239	
	(0.259)	
Error*c_P_news	0.037	
	(0.036)	
Constant	0.600 ***	17.307 ***
	(0.042)	(0.689)
BP test for homoscedasticity		113.10 ***
Observations	5207	5207

Note: Column 1 is the first stage regression; variables are preceded by C_ to centralize them (e.g., c_Gender represents the centralized processing of the variable gender). Column 2 is the second stage regression. Standard errors in parentheses (clustered at the county level). *** *p* < 0.01, ** *p* < 0.05, * *p* < 0.1.

**Table 6:** Regression of thresholds for age and family income.

Variables	(1)	(2)	(3)	(4)
Thresholds	Region 1	Region 2	Region 1	Region 2
Age	<51	>51		
Income			<40,000	>40,000
*B_drink*	−0.489 ***	−0.271 **	−0.651 ***	−0.178
	(0.120)	(0.125)	(0.119)	(0.124)
Control variable	Yes	Yes	Yes	Yes
Constant	16.577 ***	17.404 ***	17.212 ***	18.259 ***
	(0.685)	(0.418)	(0.410)	(0.595)
Observations	5207	5207	5207	5207

Note: Columns 1 and 2 show the age-threshold regression of the effect of alcohol consumption on trust; the threshold value is 51. Columns 3 and 4 show the income-threshold regression of the effect of alcohol consumption on trust; the threshold value is CNY 40,000. Standard errors in parentheses (clustered at the county level). *** *p* < 0.01, ** *p* < 0.05, * *p* < 0.1.

## Data Availability

The data sets used and/or analyzed during the current study are available from the corresponding author on reasonable request.

## References

[1] Heath D.B. (1995). International Handbook on Alcohol and Culture.

[2] Meyer E. (2014). The Culture Map: Breaking through the Invisible Boundaries of Global Business.

[3] Fang Z., Jin X. (2019). Chinese Alcohol Culture and Corporate Rent-seeking Behavior. J. Appl. Financ. Bank..

[4] Wei H., Derson Y., Shuiyuan X., Lingjiang L., Yalin Z. (1999). Alcohol consumption and alcohol-related problems: Chinese experience from six area samples, 1994. Addiction.

[5] Chua R.Y., Morris M.W., Ingram P. (2009). Guanxi vs. networking: Distinctive configurations of affect-and cognition-based trust in the networks of Chinese vs. American managers. J. Int. Bus. Stud..

[6] Huang C., Wang J., Xu L. Drinking into Friends: Alcohol Drinking Culture and CEO Social Connections. Available at SSRN 3501300 2019. https://papers.ssrn.com/sol3/papers.cfm?abstract_id=3501300.

[7] Hao W., Chen H., Su Z. (2005). China: Alcohol today. Addiction.

[8] Buckman J.F., Bates M.E., Cisler R.A. (2007). Social networks and their influence on drinking behaviors: Differences related to cognitive impairment in clients receiving alcoholism treatment. J. Stud. Alcohol Drugs.

[9] Stout R.L., Kelly J.F., Magill M., Pagano M.E. (2012). Association between Social Influences and Drinking Outcomes Across Three Years. J. Stud. Alcohol Drugs.

[10] Matsuda M., Osilla K.C., Kennedy D.P., Paddock S.M. (2021). Longitudinal effects of social network changes on drinking outcomes for individuals with a first-time DUI. J. Subst. Abus. Treat..

[11] Kim S., Spilman S.L., Liao D.H., Sacco P., Moore A.A. (2018). Social networks and alcohol use among older adults: A comparison with middle-aged adults. Aging Ment. Health.

[12] Plant M.A., Plant M.L., Miller P., Gmel G., Kuntsche S. (2009). The Social Consequences of Binge Drinking: A Comparison of Young Adults in Six European Countries. J. Addict. Dis..

[13] Ahnquist J., Lindström M., Wamala S.P. (2008). Institutional trust and alcohol consumption in Sweden: The Swedish National Public Health Survey 2006. BMC Public Health.

[14] Åslund C., Nilsson K.W. (2013). Social capital in relation to alcohol consumption, smoking, and illicit drug use among adolescents: A cross-sectional study in Sweden. Int. J. Equity Health.

[15] Tutenges S., Sandberg S. (2013). Intoxicating stories: The characteristics, contexts and implications of drinking stories among Danish youth. Int. J. Drug Policy.

[16] Nie X., Zhu Y., Fu H., Dai J., Gao J. (2018). The “Dark Side” Effects of Social Capital on Harmful Drinking among Chinese Community Residents: A Multilevel Study. Int. J. Environ. Res. Public Health.

[17] Gao J., Weaver S.R., Fua H., Pan Z. (2014). Does workplace social capital associate with hazardous drinking among Chinese rural-urban migrant workers?. PLoS ONE.

[18] Chen W.-Q., Wong T.W., Yu I.T.-S. (2008). Association of occupational stress and social support with health-related behaviors among Chinese offshore oil workers. J. Occup. Health.

[19] Chuang Y.-C., Chuang K.-Y. (2008). Gender differences in relationships between social capital and individual smoking and drinking behavior in Taiwan. Soc. Sci. Med..

[20] Sjodin L., Livingston M., Karlsson P., Larm P., Raninen J. (2022). Associations between trust and drinking among adolescents. Drug Alcohol Rev..

[21] Takakura M. (2011). Does social trust at school affect students’ smoking and drinking behavior in Japan?. Soc. Sci. Med..

[22] Lundborg P. (2005). Social capital and substance use among Swedish adolescents-an explorative study. Soc. Sci. Med..

[23] Takakura M. (2015). Relations of participation in organized activities to smoking and drinking among Japanese youth: Contextual effects of structural social capital in high school. Int. J. Public Health.

[24] Reid A.E., Carey K.B., Merrill J.E., Carey M.P. (2015). Social Network Influences on Initiation and Maintenance of Reduced Drinking Among College Students. J. Consult. Clin. Psychol..

[25] Lee I.C., Ting T.T., Chen D.R., Tseng F.Y., Chen W.J., Chen C.Y. (2015). Peers and social network on alcohol drinking through early adolescence in Taiwan. Drug Alcohol Depend..

[26] Cruz J.E., Emery R.E., Turkheimer E. (2012). Peer Network Drinking Predicts Increased Alcohol Use from Adolescence to Early Adulthood After Controlling for Genetic and Shared Environmental Selection. Dev. Psychol..

[27] Fujimoto K., Valente T.W. (2013). Alcohol Peer Influence of Participating in Organized School Activities: A Network Approach. Health Psychol..

[28] Overbeek G., Bot S.M., Meeus W.H.J., Sentse M., Knibbe R.A., Engels R. (2011). Where It’s At! The Role of Best Friends and Peer Group Members in Young Adults’ Alcohol Use. J. Res. Adolesc..

[29] Jacob L., Smith L., Armstrong N.C., Yakkundi A., Barnett Y., Butler L., McDermott D.T., Koyanagi A., Shin J.I., Meyer J. (2021). Alcohol use and mental health during COVID-19 lockdown: A cross-sectional study in a sample of UK adults. Drug Alcohol Depend..

[30] Witkiewitz K., Kranzler H.R., Hallgren K.A., O’Malley S.S., Falk D.E., Litten R.Z., Hasin D.S., Mann K.F., Anton R.F. (2018). Drinking risk level reductions associated with improvements in physical health and quality of life among individuals with alcohol use disorder. Alcohol. Clin. Exp. Res..

[31] Rumgay H., Shield K., Charvat H., Ferrari P., Sornpaisarn B., Obot I., Islami F., Lemmens V.E.P.P., Rehm J., Soerjomataram I. (2021). Global burden of cancer in 2020 attributable to alcohol consumption: A population-based study. Lancet Oncol..

[32] Nugroho T.W., Hanani N., Toiba H., Sujarwo S. (2022). Promoting Subjective Well-Being among Rural and Urban Residents in Indonesia: Does Social Capital Matter?. Sustainability.

[33] Leonardi R., Nanetti R.Y., Putnam R.D. (2001). Making Democracy Work: Civic Traditions in Modern Italy.

[34] Stevrin P. (1998). Tillitskrisen: Om Tillit, Misstro och Kontroll i det Framväxande Informationssamhället.

[35] Cho Y.J., Lee J.W. (2011). Perceived Trustworthiness of Supervisors, Employee Satisfaction and Cooperation. Public Manag. Rev..

[36] Hasche N., Höglund L., Mårtensson M. (2020). Intra-organizational trust in public organizations–the study of interpersonal trust in both vertical and horizontal relationships from a bidirectional perspective. Public Manag. Rev..

[37] Seid A.K., Hesse M., Bloomfield K. (2016). Make it another for me and my mates’: Does social capital encourage risky drinking among the Danish general population?. Scand. J. Public Health.

[38] Seid A.K. (2016). Social interactions, trust and risky alcohol consumption. Health Econ. Rev..

[39] Sayette M.A., Creswell K.G., Dimoff J.D., Fairbairn C.E., Cohn J.F., Heckman B.W., Kirchner T.R., Levine J.M., Moreland R.L. (2012). Alcohol and group formation: A multimodal investigation of the effects of alcohol on emotion and social bonding. Psychol. Sci..

[40] Kirchner T.R., Sayette M.A., Cohn J.F., Moreland R.L., Levine J.M. (2006). Effects of alcohol on group formation among male social drinkers. J. Stud. Alcohol.

[41] Dunbar R.I.M., Launay J., Wlodarski R., Robertson C., Pearce E., Carney J., MacCarron P. (2017). Functional Benefits of (Modest) Alcohol Consumption. Adapt. Hum. Behav. Physiol..

[42] De Visser R.O., McDonnell E.J. (2012). ‘That’s, O.K. He’s a guy’: A mixed-methods study of gender double-standards for alcohol use. Psychol. Health.

[43] Gordon R., Heim D., MacAskill S. (2012). Rethinking drinking cultures: A review of drinking cultures and a reconstructed dimensional approach. Public Health.

[44] Finkle A., Shin D. (2014). An Economic Theory of Workaholics and Alcoholics. Econ. Inq..

[45] Haucap J., Herr A. (2014). A note on social drinking: In Vino Veritas. Eur. J. Law Econ..

[46] Frank B., Haucap J., Herr A. (2014). Social Drinking Versus Administering Alcohol. Econ. Inq..

[47] Bray J.W. (2005). Alcohol use, human capital, and wages. J. Labor Econ..

[48] Ziebarth N.R., Grabka M.M. (2009). In vino pecunia? The association between beverage-specific drinking behavior and wages. J. Labor Res..

[49] Groh D.R., Jason L.A., Keys C.B. (2008). Social network variables in alcoholics anonymous: A literature review. Clin. Psychol. Rev..

[50] MacLean S. (2016). Alcohol and the Constitution of Friendship for Young Adults. Sociology.

[51] Szmigin I., Griffin C., Mistral W., Bengry-Howell A., Weale L., Hackley C. (2008). Re-framing ‘binge drinking’as calculated hedonism: Empirical evidence from the UK. Int. J. Drug Policy.

[52] Törrönen J., Maunu A. (2007). Light transgression and heavy sociability: Alcohol in young adult Finns’ narratives of a night out. Addict. Res. Theory.

[53] Amenta S., Noel X., Verbanck P., Campanella S. (2013). Decoding of Emotional Components in Complex Communicative Situations (Irony) and Its Relation to Empathic Abilities in Male Chronic Alcoholics: An Issue for Treatment. Alcohol. Clin. Exp. Res..

[54] Martinotti G., Di Nicola M., Tedeschi D., Cundari S., Janiri L. (2009). Empathy Ability Is Impaired in Alcohol-Dependent Patients. Am. J. Addict..

[55] Pagano M.E., Kelly J.F., Scur M.D., Ionescu R.A., Stout R.L., Post S.G. (2013). Assessing Youth Participation in AA-Related Helping: Validity of the Service to Others in Sobriety (SOS) Questionnaire in an Adolescent Sample. Am. J. Addict..

[56] Pagano M.E., Zeltner B., Post S., Jaber J., Zywiak W., Stout R.L. (2009). Who should I help to stay sober? Helping behaviors among alcoholics who maintain long-term sobriety. Alcohol. Treat. Q..

[57] Crawford V.P., Sobel J. (1982). Strategic Information-Transmission. Econometrica.

[58] Au P.H., Lim W., Zhang J. (2022). In Vino Veritas? Communication under the influence—An experimental study. J. Econ. Behav. Organ..

[59] Lindstrom M. (2005). Social capital, the miniaturization of community and high alcohol consumption: A population-based study. Alcohol Alcohol..

[60] Fielding D., Knowles S., Robertson K. (2018). Alcohol, generosity and empathy. J. Behav. Exp. Econ..

[61] Schweitzer M.E., Gomberg L.E. (2001). The impact of alcohol on negotiator behavior: Experimental evidence 1. J. Appl. Soc. Psychol..

[62] Bregu K., Deck C., Ham L., Jahedi S. (2017). The effects of alcohol use on economic decision making. South. Econ. J..

[63] Brañas-Garza P., Mateu G., Sánchez A., Sutan A. Does Pre-Play Social Interaction Improve Negotiation Outcomes? Available at SSRN 3310341 2018. https://papers.ssrn.com/sol3/papers.cfm?abstract_id=3310341.

[64] Wang J., Houser D. (2019). An Economic Analysis of Business Drinking: Evidence from a Lab-in-the-Field Experiment. https://papers.ssrn.com/sol3/papers.cfm?abstract_id=3489451.

[65] Zhu C., Chen Q.H., Si W., Li Y.X., Chen G., Zhao Q.R. (2020). Alcohol Use and Depression: A Mendelian Randomization Study From China. Front. Genet..

[66] Glanz K. (1997). Theory at a Glance: A Guide for Health Promotion Practice: US Department of Health and Human Services, Public Health Service.

[67] Ding L., Song B., Wu C., Newman I.M., Yuen L.W., Qian L., Wang B., Zhang W., Wei P. (2021). Alcohol Use in China: Unrecorded and Recorded Bai Jiu in Three Rural Regions. Int. J. Environ. Res. Public Health.

[68] Ma Y., Gu J., Lv R. (2022). Job Satisfaction and Alcohol Consumption: Empirical Evidence from China. Int. J. Environ. Res. Public Health.

[69] Meng T.G., Chen H. (2014). A multilevel analysis of social capital and self-rated health: Evidence from China. Health Place.

[70] Jiang J., Wang P. (2019). Is Linking Social Capital More Beneficial to the Health Promotion of the Poor? Evidence from China. Soc. Indic. Res..

[71] Lewbel A. (2012). Using Heteroscedasticity to Identify and Estimate Mismeasured and Endogenous Regressor Models. J. Bus. Econ. Stat..

[72] Cochrane J., Chen H.H., Conigrave K.M., Hao W. (2003). Alcohol use in China. Alcohol Alcohol..

[73] Wang J.X., Rao Y.L., Houser D.E. (2017). An experimental analysis of acquired impulse control among adult humans intolerant to alcohol. Proc. Natl. Acad. Sci. USA.

[74] Hansen B.E. (2000). Sample splitting and threshold estimation. Econometrica.

[75] Cooper M.L. (1994). Motivations for alcohol use among adolescents: Development and validation of a four-factor model. Psychol. Assess..

[76] Gonzalez V.M., Collins R.L., Bradizza C.M. (2009). Solitary and social heavy drinking, suicidal ideation, and drinking motives in underage college drinkers. Addict. Behav..

[77] Christiansen M., Vik P.W., Jarchow A. (2002). College student heavy drinking in social contexts versus alone. Addict. Behav..

[78] Creswell K.G. (2021). Drinking together and drinking alone: A social-contextual framework for examining risk for alcohol use disorder. Curr. Dir. Psychol. Sci..

[79] Yu M.M., Gu L.B., Jiao W.J., Xia H.Z., Wang W.R. (2019). Predictors of self-neglect among community-dwelling older adults living alone in China. Geriatr. Nurs..

[80] Steele C.M., Josephs R.A. (1990). Alcohol Myopia-Its Prized and Dangerous Effects. Am. Psychol..

[81] Macdonald T.K., Zanna M.P., Fong G.T. (1995). Decision-Making in Altered States-Effects of Alcohol on Attitudes toward Drinking and Driving. J. Personal. Soc. Psychol..

[82] Denton K., Krebs D. (1990). From the Scene to the Crime-the Effect of Alcohol and Social-Context on Moral Judgment. J. Personal. Soc. Psychol..

[83] Wynne L.A. (2020). ‘Just Right’: Balance, tranquility, and drunkenness in rural Yucatan. Glob. Public Health.

[84] Blume S.B. (1986). Women and Alcohol—A Review. JAMA.

[85] Ma L., Christensen T. (2018). Government Trust, Social Trust, and Citizens’ Risk Concerns: Evidence from Crisis Management in China. Public Perform. Manag. Rev..

[86] Christensen T., Fimreite A.L., Laegreid P. (2011). Crisis Management: The Perceptions of Citizens and Civil Servants in Norway. Adm. Soc..

[87] Zhang C. (2018). Family support or social support? The role of clan culture. J. Popul. Econ..

[88] Brandt M.J., Wetherell G., Henry P.J. (2015). Changes in Income Predict Change in Social Trust: A Longitudinal Analysis. Political Psychol..

[89] Ananyev M., Guriev S. (2019). Effect of Income on Trust: Evidence from the 2009 Economic Crisis in Russia. Econ. J..

